# Temperature management following cardiac arrest: introducing a protocol improves compliance with targets

**DOI:** 10.1186/cc13689

**Published:** 2014-03-17

**Authors:** P Creber, G Talling, M Oram

**Affiliations:** 1 Cheltenham General Hospital, Cheltenham, UK

## Introduction

We audited the achievement of therapeutic hypothermia (TH) before and after the introduction of a cooling protocol. Instituting TH is recommended following the return of spontaneous circulation (ROSC) for many patients who survive a cardiac arrest [1,2]. The key intervention may be the avoidance of hyperthermia rather than cooling [3].

## Methods

We conducted a chart review of all patients admitted to the Department of Critical Care (DCC) at our hospital following cardiac arrest over 2 years in 2010 to 2012 (Group 1). We recorded compliance with key recommendations produced by the Royal College of Anaesthetists [4] although we defined post-ROSC hyperthermia as >37.2°C rather than >38°C. A TH protocol was designed and personnel in the emergency department and DCC educated as to its use. Recommended practice was the infusion of cold i.v. normal saline (1 to 2 l) followed by the use of an intravascular cooling device (Alsius CoolGard™). Data collection was then undertaken after introduction of the protocol for all patients admitted to the DCC following cardiac arrest in November 2012 to 2013 (Group 2).

## Results

Forty-three patients were admitted in Group 1, 28 in Group 2. Of these, 42% in both groups were following out-of-hospital (OOH) VF arrests. Cooling was attempted in 88% and 82% of OOH VF patients respectively. For patients with either in-hospital or non-VF/ VT cardiac arrests, the numbers cooled were 16% and 12.5%. Cooling initiation within 1 hour increased from 27 to 50%. Achievement of a target temperature of 32 to 34°C within 4 hours of ROSC was 55% and 50% respectively. Target maintenance for 12 to 24 hours after ROSC increased 79% to 100%. Avoidance of hypothermia <31°C for 48 hours after ROSC improved 95% to 100%. Slow rewarming at 0.25 to 0.5°C/ hour to 37°C was achieved in 76% and 90%. Avoidance of temperature >37.2°C for 48 hours after ROSC increased 84 to 100%. Of the patients cooled, survival with good neurological outcome was achieved in 52% in Group 1 and 88% in Group 2.

## Conclusion

The institution of a temperature management protocol improved compliance with recommended goals, both in achieving hypothermia and in the avoidance of hyperthermia.

## References

[B1] Nolan JP ERC Guidelines 2010 Resuscitation 2010 81 1219 1276 10.1016/j.resuscitation.2010.08.021 20956052

[B2] HACA Study Group N Engl J Med 2002 346 549 55611856793

[B3] Nielsen N N Engl J Med 2013 369 2197 2206 10.1056/NEJMoa1310519 24237006

[B4] Nolan J Colvin J, Peden C Implementation of therapeutic hypothermia Raising the Standard In Compendium of Audit Recipes 2012 3 Royal College of Anaesthetists 200 201

